# Reconciling the Theory and the Practice of the Rule of Law in the European Union Measuring the Rule of Law

**DOI:** 10.1007/s40803-022-00171-z

**Published:** 2022-04-13

**Authors:** Julinda Beqiraj, Lucy Moxham

**Affiliations:** Bingham Center for the Rule of Law, London, UK

## Abstract

The Rule of Law has gained global appeal and recognition, and is one of the fundamental values upon which the European Union (EU) is based, as set out in Article 2 of the Treaty on European Union. In Sect. 1, we briefly consider the main elements of the Rule of Law, in particular those definitions used in the context of the EU, the Council of Europe and the United Nations (UN). Whilst acknowledging that there are national differences among EU Member States, there is broad consensus around the core meaning of the Rule of Law. These definitional issues help frame the discussion that follows on measuring the Rule of Law. In Sect. 2, we outline some general considerations regarding the rationale for measuring the Rule of Law, followed by some specific examples of measurement tools in the context of the Council of Europe (e.g., the European Commission for the Efficiency of Justice (CEPEJ) tools on evaluating the efficiency and quality of European judicial systems, and the Venice Commission Rule of Law Checklist); the EU (e.g., the EU Justice Scoreboard, the Special Eurobarometer on the Rule of Law, and the Rule of Law Report); and the UN (e.g., the Sustainable Development Goals). In Sect. 3, we consider current Rule of Law trends in light of results from a range of datasets (including the Varieties of Democracy (V-Dem) indices, the Democracy Barometer, the Bertelsmann Stiftung’s Transformation Index (BTI), and the World Justice Project’s Rule of Law Index).

## Introduction: Rule of Law Definitions and Components

General formulations and differing interpretations of the Rule of Law have led some to question its significance and usefulness as a concept.^1^ However, there is now broad consensus around its core meaning and once the main elements of the Rule of Law have been identified, it attains a very practical significance and progress towards its implementation can be measured.^2^ In this Section, we briefly consider the core components of the Rule of Law, in particular those definitions used in the context of the EU, the Council of Europe and the UN.

In his influential book ‘The Rule of Law’, the British judge Lord Bingham set out a clear definition of the Rule of Law: “that all persons and authorities within the state, whether public or private, should be bound by and entitled to the benefit of laws publicly made, taking effect (generally) in the future and publicly administered in the courts”.^3^ Lord Bingham went on to identify eight key ingredients of the Rule of Law,^4^ which have been further distilled into four core Rule of Law components: legality, legal certainty, equality, and access to justice and rights.^5^ These features of the Rule of Law “promote both formal and substantive goals”.^6^ In this regard, a distinction is sometimes drawn between ‘thin’ or ‘formal’ conceptions of the Rule of Law, and ‘thick’ or ‘substantive’ understandings. In general terms, the former focus on the procedural requirements for making and applying the law, whereas ‘thicker’ definitions also include values such as fundamental rights.^7^

At a European level, the Council of Europe’s Commission for Democracy through Law, known as the Venice Commission, draws on Lord Bingham’s definition. In its 2011 ‘Report on the Rule of Law’, the Venice Commission considered the historical origins of the Rule of law and related concepts of ‘Etat de droit’ and ‘Rechtsstaat’, as well as instances of the concept of the Rule of Law at national and international levels.^8^ It was also careful to distinguish the ‘Rule of Law’ from ‘Rule by Law’, which it described as “a purely formalistic concept under which any action of a public official which is authorised by law is said to fulfil its requirements”.^9^ The Venice Commission went on to identify a consensus around six necessary elements of the Rule of Law: (1) Legality, including a transparent, accountable and democratic process for enacting law; (2) Legal certainty; (3) Prohibition of arbitrariness; (4) Access to justice before independent and impartial courts, including judicial review of administrative acts; (5) Respect for human rights; and (6) Non-discrimination and equality before the law.^10^ It concluded that the Rule of Law “does constitute a fundamental and common European standard to guide and constrain the exercise of democratic power”.^11^ Subsequently, the Venice Commission developed a ‘Rule of Law Checklist’ as a tool to assess respect for the Rule of Law, which is discussed further below.^12^

The approach of the Venice Commission was reflected in the EU Commission’s 2014 Communication, ‘A new EU Framework to strengthen the Rule of Law’ where it set out a list of Rule of Law principles which closely follow those adopted by the Venice Commission in 2011.^13^ The European Commission took a similar approach in its subsequent 2019 Communication ‘Further strengthening the Rule of Law within the Union’. It described the Rule of Law as “a well-established principle, well-defined in its core meaning” and set out the following definition: “Under the rule of law, all public powers always act within the constraints set out by law, in accordance with the values of democracy and fundamental rights, and under the control of independent and impartial courts”.^14^ The Communication also stated that the Rule of Law includes the following principles: “legality, implying a transparent, accountable, democratic and pluralistic process for enacting laws; legal certainty; prohibiting the arbitrary exercise of executive power; effective judicial protection by independent and impartial courts, effective judicial review including respect for fundamental rights; separation of powers; and equality before the law”.^15^ Again, these closely follow those set out by the Venice Commission.

The UN definition is another example of a more substantive approach to the Rule of Law. In a 2004 report, the then UN Secretary-General Kofi Annan set out a definition of the Rule of Law. For the UN system, the Rule of Law “refers to a principle of governance in which all persons, institutions and entities, public and private, including the State itself, are accountable to laws that are publicly promulgated, equally enforced and independently adjudicated, and which are consistent with international human rights norms and standards”.^16^ It requires “measures to ensure adherence to the principles of supremacy of law, equality before the law, accountability to the law, fairness in the application of the law, separation of powers, participation in decision-making, legal certainty, avoidance of arbitrariness and procedural and legal transparency”.^17^ This was echoed in the 2012 Declaration of the ‘High-Level Meeting on the Rule of Law at the National and International Levels’, the UN General Assembly’s first plenary meeting dedicated entirely to the Rule of Law,^18^ where it was emphasised that “all persons, institutions and entities, public and private, including the State itself, are accountable to just, fair and equitable laws and are entitled without any discrimination to equal protection of the law”.^19^ These UN definitions are similar in many respects to the understandings adopted by both the Council of Europe’s Venice Commission and the EU Commission.

## The Rationale for Measuring the Rule of Law

There is a vast array of measurement tools which aim to assess the state of the Rule of Law around the world. In this Section, we outline some general considerations regarding the rationale for measuring the Rule of Law, followed by some specific examples of measurement tools in the context of the Council of Europe (e.g., the European Commission for the Efficiency of Justice (CEPEJ) tools on evaluating the efficiency and quality of European judicial systems, and the Venice Commission Rule of Law Checklist); the EU (e.g., the EU Justice Scoreboard, the Special Eurobarometer on the Rule of Law, and the Rule of Law Report); and the UN (e.g., the Sustainable Development Goals).

The Rule of Law has been described as “a transnational industry that constitutes a multi-billion dollar enterprise” and it has been noted that “[t]he global effort to build [Rule of Law] has been accompanied by the development of numerous indicators that purport to measure the phenomenon”.^20^ At its core, the rationale for measuring the Rule of Law is that you cannot improve what you cannot measure. Measuring the Rule of Law allows trends within and across jurisdictions to be monitored, reveals positive developments and examples of best practice, and warns of negative shifts and upcoming crises. Such analyses can then be used by those working in the Rule of Law field. For example, in 2011, the UN developed a set of ‘Rule of Law Indicators’ focusing on criminal justice institutions in conflict and post-conflict situations.^21^ These aimed to provide information which “the United Nations, donors and development partners will be able to use to plan and monitor the impact of their efforts to build the capacity of criminal justice institutions and, more generally, strengthen the rule of law” and which national governments can use “for monitoring their own progress in developing their criminal justice institutions and strengthening the rule of law”.^22^ Similarly, in the context of the UN Sustainable Development Goals, it has been commented that “[w]ithout relevant data on social, economic and environmental challenges, countries cannot design and implement effective evidence-based policies and accurately measure, monitor and report on sustainable development, ensuring that no one is left behind”.^23^ Many, however, have pointed out the limitations and pitfalls of measuring the Rule of Law and related aspects of governance.^24^ While an evaluation of the various approaches to Rule of Law measurement is beyond the scope of this paper, we briefly consider a couple of aspects here.

First, measurement tools vary in terms of how they conceptualise and define the Rule of Law, and which aspects they select for measurement. For example, the set of indicators used in the World Justice Project’s Rule of Law Index attempts to strike a balance between ‘thick’ and ‘thin’ conceptions of the Rule of Law in order to enable it “to apply to different types of social and political systems, including those that lack many of the features that characterize democratic nations, while including sufficient substantive characteristics to render the rule of law as more than a system of rules”.^25^ In contrast, the Democracy Barometer index, developed by the Berlin Social Science Center (WZB) and the Center for Democracy Studies Aarau (ZDA) and now led by the ZDA and the Department of Political Science at the University of Zurich, which considers the Rule of Law as one of nine democratic functions that need to be fulfilled, takes a narrower, institutional focus.^26^ For example, it measures aspects of the Rule of Law such as equality before the law (i.e. impartial courts, effective independence of the judiciary, and effective impartiality of the legal system) and the quality of the legal system (i.e. judicial professionalism, confidence in the justice system, and confidence in the police).^27^ Whichever definitional approach is adopted, it is important that measurement frameworks are transparent, and clearly articulate their conceptual underpinnings so that those relying on the data can make fully informed decisions.^28^

Second, the scientific validity and reliability of Rule of Law measurement tools also varies depending, for example, on the type of sources used (e.g., primary or secondary, quantitative data, public surveys, expert assessments, document reviews etc.), and the process for aggregation and weighting.^29^ There are also concerns that measurement methods rely on data that is quickly out of date and that we need new approaches to data collection.^30^ It has been commented in this respect that “[w]hile Rule of Law indices provide invaluable, longitudinal sources of information on the evolution of the Rule of Law situation they can hardly be called timely… this creates huge problems for the users of governance and Rule of Law data who require up to date information”.^31^ A variety of approaches are used in the measurement tools discussed below. Whichever approach to measurement is taken, it is important that those presenting the data are transparent about the way in which it was collected and processed, so that its robustness and reliability can be fully assessed by end users.^32^

### Measurement in the Context of the Council of Europe

#### The CEPEJ Evaluation of Judicial Systems

Among the tools developed to measure and assess compliance with the core components of the Rule of Law, those by the Council of Europe are of essential importance. Within the Council of Europe, the European Commission for the Efficiency of Justice (CEPEJ) has developed and fine-tuned a methodology and tools for collecting, analysing and comparing data on the efficiency, quality and effectiveness of European judicial systems.^33^ Since 2004, the CEPEJ has undertaken a regular process for evaluating the judicial systems of the Council of Europe's member states and of a few other non-member states that have voluntarily agreed to join the exercise. The process is managed by the CEPEJ’s Working Group on the evaluation of judicial systems (CEPEJ-GT-EVAL),^34^ which collects, validates and processes the responses to a questionnaire of over 200 questions that is improved and updated periodically by the CEPEJ Secretariat. Responses are provided every two years by national correspondents assigned by each participating government and consist of both quantitative data and qualitative data such as context information on the legal system and explanations that enable the understanding of the figures provided. Responses are then compiled and analysed in comparative reports every two years, and are also made publicly available via the CEPEJ-STAT database.^35^ The CEPEJ tool on the evaluation of judicial systems in Europe, which is based on a specifically tailored methodology, is not directed at measuring the Rule of Law as such. However, by providing an accurate and methodologically sound assessment of justice systems in Europe, regarding their efficiency, effectiveness, and quality, it offers insights into the achievement of specific components of the Rule of Law, in particular the access to justice component. For instance, the CEPEJ-GT-EVAL periodic reports present and analyse data on budgetary allocations to the judiciary and related European trends, as well as on the salaries of judges and prosecutors and how these have developed over the years. Adequate financial resources for the judicial system are instrumental to the proper functioning and the independence of the judiciary. As stated by the Council of Europe’s Venice Commission, adequate funding is necessary to “enable the courts and judges to live up to the standards laid down in Article 6 of the European Convention on Human Rights and in national constitutions and perform their duties with the integrity and efficiency which are essential to the fostering of public confidence in justice and the rule of law”.^36^

The CEPEJ tool also reports data on the number of lawyers in the different Council of Europe member states and on the availability of free legal aid, by type of case (criminal/civil/administrative) and by stage of the dispute (in court or out of court legal advice). These aspects are essential to the Rule of Law, as highlighted in the latest CEPEJ report: “Quality of justice depends on the possibility for a litigant to be represented and for a defendant to mount his or her defence, both functions performed by a professional who is trained, competent, available, offering ethical guarantees and working at a reasonable cost.”^37^

Moreover, the CEPEJ tool reports data and assesses the main trends on the efficiency of courts and public prosecution services, using indicators developed by the CEPEJ, such as the clearance rate,^38^ the disposition time,^39^ the number of pending cases,^40^ the age of pending cases,^41^ etc. Efficiency of courts and public prosecution services is considered “one of the vital factors for upholding the rule of law and a critical component of a fair trial. It facilitates good governance, promotes the fight against corruption and builds confidence in institutions. Efficient courts and public prosecution services enable individuals to enjoy their economic and social rights and freedoms.”^42^

#### The Venice Commission’s Rule of Law Checklist

The 2016 Venice Commission Rule of Law Checklist, referenced in Sect. 1, is an instrument aimed at assessing both the presence of Rule of Law legal safeguards and the meeting of other benchmarks relating to the practice and to the implementation of laws in a given country.^43^ As earlier noted, the Checklist draws on Lord Bingham’s definition of the Rule of Law^44^ and builds on the Venice Commission’s 2011 ‘Report on the Rule of Law’,^45^ which identifies a consensus around a few necessary elements of the concept.^46^ These are further elaborated and developed into a Rule of Law Checklist, accompanied by standards, providing possible sources of verification for assessing whether the safeguards and benchmarks have been met.^47^

The Checklist aims to provide a tool for assessing a country’s Rule of Law situation from the perspective of its constitutional and legal structures, the legislation in force and the existing case-law.^48^ It focuses on five core components of the Rule of Law, namely (1) Legality; (2) Legal certainty; (3) Prevention of abuse (misuse) of powers^49^; (4) Equality before the law and non-discrimination^50^; and (5) Access to Justice.^51^ The Checklist was developed to serve as a tool for a variety of actors who may decide to carry out an assessment of the Rule of Law in their jurisdiction, including Parliaments, other State authorities, and non-governmental organisations (NGOs), as well as international organisations and regional ones such as the Council of Europe and the European Union.^52^ In this latter regard, the Checklist document has been referenced in the case law of the Court of Justice of the EU^53^ and the Venice Commission’s work has acquired increasing relevance in the EU’s accession process, especially in the definition of the yardstick of political conditionality for the aspiring EU Member States.^54^ Indeed, as clarified in the Checklist document itself, “It is not within the mandate of the Venice Commission to proceed with Rule of Law assessments in given countries on its own initiative; however, it is understood that when the Commission, upon request, deals with Rule of Law issues within the framework of the preparation of an opinion relating a given country, it will base its analysis on the parameters of the checklist within the scope of its competence”.^55^

The relevance of the Venice Commission’s Checklist stems from its innovative methodological approach to the Rule of Law, as it moves away from an abstract, academic approach and offers instead a pragmatic and operational tool at the disposal of governments, academics, NGOs and international organisations. Moreover, rather than focussing on specific components of the Rule of law, it provides a systemic tool, aimed at obtaining a comprehensive assessment of legal systems.

### Measurement in the Context of the EU

At the EU level, the Rule of Law is listed as one of the founding values of the Union in Article 2 of the Treaty on European Union (TEU), alongside human dignity, freedom, democracy, equality, and respect for human rights. Compliance with Rule of Law standards is assessed as part of the political criteria for accession to the EU (the Copenhagen criteria) and the Rule of Law must continue to be upheld while being an EU member state. To address threats to EU values, Article 7 TEU sets out that “the Council […] may determine that there is a clear risk of a serious breach by a Member State of the values referred to in Article 2” or “may determine the existence of a serious and persistent breach by a Member State of the values referred to in Article 2” and, accordingly, take preventive or sanctioning measures. Moreover, the increasing acknowledgment of the threat posed by what has been labelled Rule of Law “backsliding” has resulted in a “rapid evolution of the EU’s rule of law ‘toolbox’” and in an exponential growth, since 2012, of the measures to address such threats.^56^ The importance of Rule of Law measurement becomes apparent when considering that all these tools, including the mechanisms under Article 7 TEU, rely on a preliminary assessment of Rule of Law serious breaches or risks thereof. This also explains the relevance of and references to the Council of Europe’s definitions and measurement tools in the EU’s mechanisms and measurement efforts.^57^

#### The EU Justice Scoreboard

Among the EU’s measurement tools, the European Commission has issued an annual EU Justice Scoreboard since 2013,^58^ which presents comparable data on the independence, quality and efficiency of national justice systems of the EU Member States. These three components are considered essential parameters of effective justice systems that uphold the Rule of Law.^59^ The EU Justice Scoreboard is part of the ‘Rule of Law toolbox’ that the EU has been building to help monitor and uphold the Rule of Law in the Member States.

As regards the independence component, among other things, the Justice Scoreboard presents data on the independence of courts and judges, as perceived by the general public and by companies. Sources are the Eurobarometer^60^ and the Global Competitiveness report of the World Economic Forum.^61^ The 2021 Scoreboard, for instance, contains information about the rules on the appointment of Supreme Court judges, their individual performance evaluation, and rules on promotion, as well as safeguards relating to the functioning of national prosecution services.^62^

As to the parts of the Scoreboard on efficiency and quality of justice, the largely quantitative data collection organised by the Council of Europe’s CEPEJ provides the basis for the European Union’s Justice Scoreboard. Instead of every two years, data is collected by the CEPEJ and according to the CEPEJ’s methodology every year and the Scoreboard is published yearly, compiling data only from the European Union countries. Data covers information on the number of judges, court personnel, resources, digitalisation and court performance (i.e., the CEPEJ efficiency indicators described above, such as the clearance rate and the disposition time).^63^

#### The 2019 Special Eurobarometer on the Rule of Law

In addition to the above-mentioned Eurobarometer surveys on the perceived independence of justice systems, in April 2019, a Special Eurobarometer survey on the Rule of Law in all EU Member States was undertaken at the request of the Directorate-General for Justice and Consumers of the European Commission.^64^ Rather than measuring and evaluating the state of the Rule of Law in the different EU countries, the survey aims to assess how important the Rule of Law is for EU citizens, whether there is a perceived need for improvement and what is the citizens’ knowledge of EU values.^65^ The report and its findings are based on interviews conducted with 27,655 respondents from 28 EU Member States (at that time).^66^

As regards the meaning of the Rule of Law for the purposes of the Special Eurobarometer, the survey breaks down the concept into 17 principles. These principles are grouped into three thematic areas, namely (1) legality, legal certainty, equality before the law and separation of powers; (2) prohibition of arbitrariness and penalties for corruption; and (3) effective judicial protection by independent courts.^67^ These closely follow and represent a distilled version of the elements set out in the Venice Commission’s Rule of Law Checklist.

The survey first looks at the importance of the Rule of Law and whether there is a need to improve respect for the Rule of Law and in which ways. Secondly, the survey asks respondents to assess the importance of respect for the Rule of Law throughout the EU, namely in other Member States and in the Union more generally. Finally, the survey asks respondents to assess how well-informed they are about the fundamental values of the EU. The results of the Special Eurobarometer survey “show overwhelming support for the Rule of Law, with limited differences between Member States”.^68^ They also indicate a significant consistency of opinion with respect to the 17 principles of the Rule of Law that are covered by the survey. The “importance of the key principles of the Rule of Law was recognised by over 80% of citizens in all Member States”.^69^ The Eurobarometer also “underlined that Europeans consider it important that the Rule of Law applies throughout the EU, with 89% supporting the need for the Rule of Law to be respected in all other EU Member States”.^70^ It further “revealed that over half of Europeans do not feel sufficiently informed about the EU’s fundamental values”.^71^

#### The Rule of Law Report

In July 2019, the European Commission issued a communication addressed to the other EU institutions, entitled ‘Strengthening the Rule of Law within the Union: A Blueprint for Action’.^72^ The Blueprint for Action aims to reinforce the existing Rule of Law toolbox set out in the April 2019 Communication of the Commission^73^ following three paths: promotion, prevention and (effective common) response.^74^ Under the prevention prong, the Commission commits to deepening the EU’s monitoring of Rule of Law developments in the Member States through a Rule of Law Review Cycle, the results of which would be published in an annual Rule of Law Report summarising the situation in the Member States.^75^ It would also further develop the EU Justice Scoreboard to improve its coverage of Rule of Law related areas.^76^

The Blueprint for Action document explains that the review cycle would cover all the different components of the Rule of Law, including systemic problems with the process for enacting laws, lack of effective judicial protection by independent and impartial courts, challenges to the separation of powers, the ability of Member States to fight corruption, issues regarding media pluralism and elections, and also issues related to the effective enforcement of EU law.^77^ The review cycle would concern all Member States and would be “more intense in Member States where risks of regression, or particular weaknesses, have been identified”.^78^ Sources for the review cycle are data gathered by the bodies of the Council of Europe, the Organisation for Security and Cooperation in Europe, the Organisation for Economic Cooperation and Development, and the EU’s Fundamental Rights Agency,^79^ and the Annual Rule of Law Report would provide a “synthesis of significant developments in the Members States and at EU level, including the case law of the European Court of Justice, and other relevant information, such as relevant parts of the EU Justice Scoreboard and of the European Semester country reports”.^80^

The 2020 Rule of Law Report that was published on 30 September 2020 contains on the first page a very clear definition of the Rule of Law inspired by the Venice Commission’s Rule of Law checklist of 2011 and the Commission’s 2014 definition.^81^ A selection of four specific subject matters (justice systems, anti-corruption, media pluralism and freedom, and separation of powers/checks and balances) allows for some comparability between Member States and provides an overview of where Europe stands with regard to the Rule of Law. However, it has been observed that one of the main weaknesses of the report is that “when it comes to systemic breaches, the Report fails to give the necessary context, and to connect the dots. Although it does give a devastating picture of notorious Rule of Law violators, the scale and especially the systemic nature of the problem of state capture is not made clear to the reader”.^82^ Another important deficiency is that “the document does not foresee remedies, it solely aims to give an assessment of the Rule of Law situation in the Member States, which may later feed into procedures that are designed to respond to Rule of Law violations”.^83^

The 2021 Rule of Law Report covers the same four subject matters as the 2020 Report and also develops further the analysis on the impact and challenges brought by the COVID-19 pandemic in relation to each of them.^84^ As regards judicial independence, the Report notes that “Eurobarometer surveys conducted among both the general public and businesses in 2021 show that, compared to 2020, the same Member States continue to cluster around the higher [Austria, Finland, Germany, the Netherlands, and Luxembourg, above 75%] and lower [Croatia, Poland and Slovakia, below 30%] end of the scale of perceived judicial independence”.^85^ The Report also warns about “[s]erious structural concerns” regarding judicial independence that exist in a few Member States and have deepened, such as in Poland and Hungary, and that challenges remain in other Member States such as Bulgaria and Slovakia.^86^ As in the previous report, the conclusions do not identify specific remedies but rather use general language on the Member States’ “continued engagement and cooperation and a willingness to enter into a dialogue on sensitive issues”.^87^

### Measurement in the Context of the UN

#### The UN Sustainable Development Goals and the Rule of Law

The UN Sustainable Development Goals (SDGs) are described as “a universal call to action to end poverty, protect the planet and improve the lives and prospects of everyone, everywhere”.^88^ The 2030 Agenda for Sustainable Development was adopted by all UN Member States in September 2015 and included 17 Goals and 169 related Targets.^89^

The Rule of Law is seen in the 2030 Agenda as both an outcome of development and as integral to the achievement of the SDGs as a whole. While the Rule of Law is not explicitly mentioned in the Goals themselves, the new Agenda recognises “the need to build peaceful, just and inclusive societies that provide equal access to justice and that are based on respect for human rights (including the right to development), on effective rule of law and good governance at all levels and on transparent, effective and accountable institutions”.^90^ In addition, access to justice issues are included in Goal 16 which aims to “Promote peaceful and inclusive societies for sustainable development, provide access to justice for all and build effective, accountable and inclusive institutions at all levels”.

Within Goal 16, several Targets are relevant from a Rule of Law perspective, in particular Target 16.3 (Promote the rule of law at the national and international levels and ensure equal access to justice for all). Other Goal 16 Targets which are relevant for the Rule of Law include, for example, Target 16.6 (Develop effective, accountable and transparent institutions at all levels); Target 16.7 (Ensure responsive, inclusive, participatory and representative decision-making at all levels); Target 16.10 (Ensure public access to information and protect fundamental freedoms, in accordance with national legislation and international agreements); and Target 16.B (Promote and enforce non-discriminatory laws and policies for sustainable development). There are also links to the Rule of Law in many of the other Goals such as SDG 5 (Achieve gender equality and empower all women and girls); SDG 8 (Promote sustained, inclusive and sustainable economic growth, full and productive employment and decent work for all); and SDG 10 (Reduce inequality within and among countries).

#### The SDG Indicators and the Rule of Law

In terms of measuring progress, the Goals and their related Targets are underpinned by a global indicator framework, which was developed by the Inter-Agency and Expert Group on SDG Indicators (IAEG-SDGs), through an open, inclusive and transparent process, and which was adopted by the UN General Assembly in July 2017.^91^ The global indicators are refined each year and there was a comprehensive review in 2020, with another review due in 2025.^92^

Focusing on the Rule of Law and in particular Target 16.3, there were originally two indicators for this Target: “16.3.1 Proportion of victims of violence in the previous 12 months who reported their victimization to competent authorities or other officially recognized conflict resolution mechanisms” and “16.3.2 Unsentenced detainees as a proportion of overall prison population”.^93^ These indicators both focus on criminal justice and, as has been commented, “fail to take into consideration legal representation issues, access to justice in sectors other than criminal, whether formal or informal” and “tell little about the accessibility, affordability, equality and fairness of the justice system or the degree of public confidence in it”.^94^

A third indicator for Target 16.3 was subsequently added as part of the 2020 Comprehensive Review.^95^ The additional indicator 16.3.3 on access to justice is worded as follows: “Proportion of the population who have experienced a dispute in the past two years and who accessed a formal or informal dispute resolution mechanism, by type of mechanism”.^96^ The new indicator will go some way to addressing limitations of the original indicator framework for Target 16.3.^97^ However, as was remarked about the selection process for Goal 16 indicators and Target 16.3 in particular, “[i]t is hard to imagine how one or even a handful of indicators would be able to capture such complex, multi-faceted reality both at the national and international levels for the whole world”.^98^ The commentators cautioned in this regard, “[s]tretching good indicators beyond their inherent limitations may lead to inadequate policy-making at a global scale”.^99^

Regional and national level indicators developed by the UN Member States will complement the global indicator framework and will provide additional opportunities for meaningful measurement of the Rule of Law.^100^ For example, Eurostat (the statistical office of the EU) has coordinated the development of the EU SDG indicator set and produces regular monitoring reports on progress in an EU context.^101^ However, some have expressed concern that regional and national initiatives are undermining global efforts, rather than complementing them.^102^

Based on the global indicator framework, two key reports are published each year ahead of the UN High-Level Political Forum on Sustainable Development (HLPF), which has a central role in follow-up and review of the SDGs at the international level.^103^ The UN Secretary-General releases an annual ‘Progress Report’ on the SDGs,^104^ which is accompanied by a ‘Statistical Annex’.^105^ An annual ‘Sustainable Development Goals Report’ is also released.^106^ These two reports are accompanied by a ‘Global SDG Indicators Database’,^107^ which is a database of available global, regional and country data and metadata for the SDG indicators.^108^ Both of these reports are based on the latest available data on selected indicators in the global indicator framework.^109^ The UN Statistics Division (UNSD) receives data from “custodian agencies”, which are the agencies responsible for compiling internationally comparable data for specific indicators.^110^ For example, for indicators 16.3.1 and 16.3.2, the custodian agency is the UN Office on Drugs and Crime (UNODC), and for indicator 16.3.3 the custodian agencies are the UNODC, the UN Development Programme (UNDP), and the Organisation for Economic Cooperation and Development (OECD).^111^

#### Measuring Progress Towards the SDGs and Data Challenges

Data is, therefore, at the heart of the 2030 Agenda and it has been commented that “SDG implementation will be measured and driven through monitoring, based on the indicators”.^112^ Indeed, Target 17.18 specifically addresses this issue and by 2020 aims to enhance capacity-building support “to increase significantly the availability of high-quality, timely and reliable data disaggregated by income, gender, age, race, ethnicity, migratory status, disability, geographic location and other characteristics relevant in national contexts”.

However, there are significant data challenges and, as a result, it has been observed that “the [Secretary-General’s] annual reports are as much a reflection on the availability of globally comparable data as they are a snapshot of SDG progress”.^113^ Against this background, while noting the progress made in terms of the availability of internationally comparable data, the ‘Sustainable Development Goals Report 2021’ emphasises that “big data gaps still exist in all areas of the SDGs in terms of geographic coverage, timeliness and the level of disaggregation required”.^114^ Of particular relevance for present purposes, the 2021 Report notes that “[c]ountry-level data deficits are also significant in areas related to… peace, justice and strong institutions (Goal 16)”.^115^ In addition, the previous ‘Sustainable Development Goals Report 2020’ emphasised that the COVID-19 pandemic has not only impacted the realisation of the SDGs themselves but is also making data inequalities worse by “disrupting routine operations throughout the global statistical and data system, with delays in planned censuses, surveys and other data programmes”.^116^ This is echoed in the 2021 Report which warns that “lockdown measures implemented to control the spread of COVID-19 have hindered data collection efforts for much of 2020, widening gaps in the capacity of countries to report on many of the indicators”.^117^ Recent changes to the global indicator framework, such as the addition of new indicator 16.3.3, discussed above, have also impacted on the data that is needed for SDG reporting. A further challenge is that SDG monitoring requires data in areas not traditionally covered by official statistics.^118^

In Sect. 2, we have considered some specific examples of measurement tools in the context of the Council of Europe, the EU and the UN. In Sect. 3, we consider current Rule of Law trends in light of results from a range of Rule of Law datasets.

## Rule of Law Trends: Global Outlook

In addition to the measurement tools outlined in the previous sections, there is a variety of indices aimed at measuring the quality and strength of the Rule of Law in countries around the world. We address below some of the most important ones and we also highlight some of their main findings.

### Varieties of Democracy (V-Dem) Indices

The Varieties of Democracy (V-Dem) is one of the largest social science data collection projects on democracy and its headquarters are in Sweden.^119^ A team of over 50 social scientists on six continents, working with more than 3,200 country experts, have created a database containing over 28.4 million data points, covering 202 countries from 1789 to 2020, with annual updates to follow.^120^ Half of the indicators in the V-Dem dataset are based on factual information obtainable from official documents, such as constitutions and government records, and the remainder consist of more subjective assessments. Here, we look at the V-Dem Rule of Law and Liberal component indices.

The V-Dem Rule of Law index aggregates indicators such as the government’s compliance with court decisions, court independence, whether public authorities and public administration are impartial and respect the country’s constitution, transparent laws with predictable enforcement, access to justice, and judicial accountability for misconduct and corruption.^121^ RECONNECT research has found that, at the global level, this index essentially remained stable throughout the period 2000–2018.^122^ At the national level, the most significant drops were recorded in Turkey (−  0.58), Burundi (− 0.45), and Thailand (− 0.3), while notable improvements were observed in Tunisia (+ 0.69), Peru (+ 0.61), and Georgia (+ 0.61). In the EU, at a country level, the most significant decline in the Rule of Law over the period 2000–2018 was recorded in Hungary (− 0.18), followed by Bulgaria (− 0.13) and Poland (− 0.12). Interestingly, Romania experienced a significant improvement of the Rule of Law until 2016, followed by a marked decline.

The V-Dem Liberal Democracy Index also captures some core features of the Rule of Law such as equality before the law and individual liberties, judicial constraints on the executive, and legislative constraints on the executive. The latest 2021 report, which considers the state of liberal democracy in 2020, concludes that while the world is more democratic than it was in the 1970s and 1980s, a steep global decline in liberal democracy has taken place during the past 10 years and continued in 2020, this being especially prominent in the Asia–Pacific region, Eastern Europe and Central Asia, and Latin America.^123^

To look at this in more detail, the country scores for 2017 showed a decrease for the USA, Brazil, Poland, Serbia, Macedonia, Ukraine, and Turkey among others.^124^ Some of these countries, namely the USA, Brazil, Poland, Serbia and Turkey, showed further decline also in the 2018, 2019, and 2020 measurements.^125^ In 2020, autocracies were home to 68% of the world population, a sharp increase since 2010 (48%), and liberal democracies diminished over the past decade from 41 countries to 32, with a population share of only 14%.^126^ The 2021 report also finds that “States in Eastern Europe such as Hungary, Poland, and Serbia have continued their downward decline after continued assaults on the judiciary and restrictions on the media and civil society”.^127^

In the top ten list of major decliners in liberal democracy, four countries are in Europe. Notably, since 2019, the EU has a non-democratic state as a member: Hungary being now classified as an electoral authoritarian regime. In 2020, “while Hungary’s ongoing autocratization is still conspicuous, Poland has taken over the dubious first position with a dramatic 34 percentage points decline on the LDI, most of which has occurred since 2015”.^128^ The table below classifies countries by regime types (Closed Autocracy, Electoral Autocracy, Electoral Democracy and Liberal Democracy) and shows the regime type change in the last 10 years.

**Figure:**
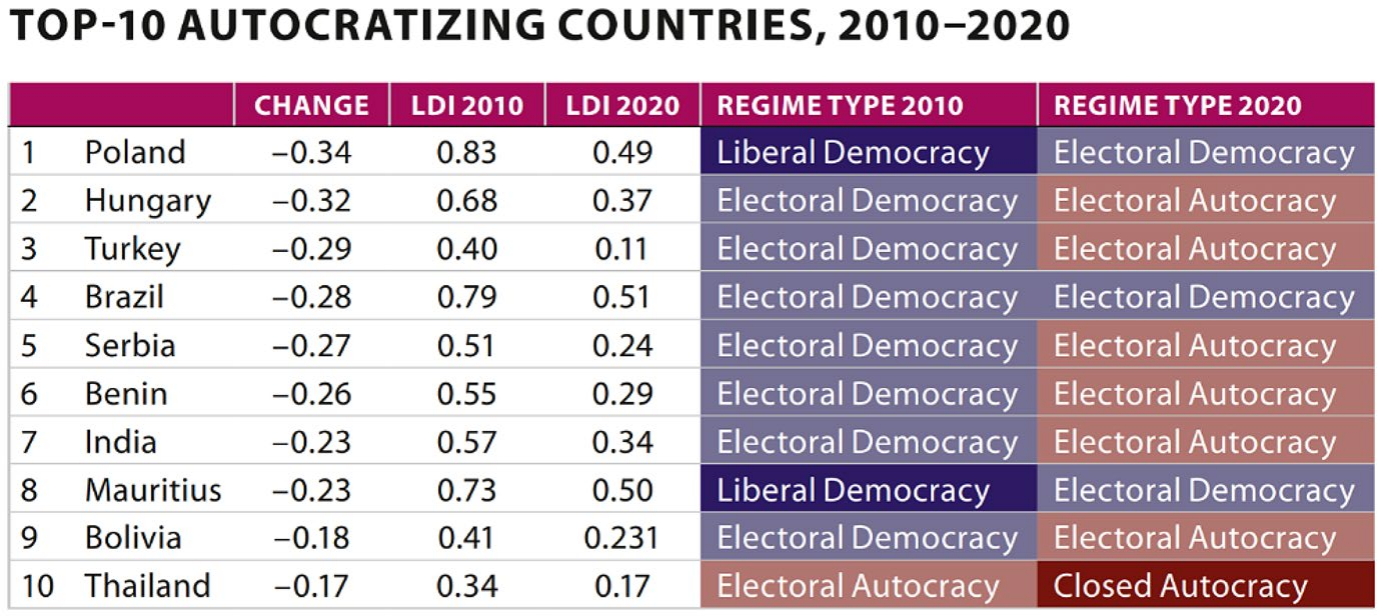
Source: V-DEM Annual Democracy Report 2021 (V-Dem Annual Democracy Report 2021 ‘Autocratization Turns Viral’, noted above, at page 16)

Similarly, the figures below show a worsening in key democracy aspects, including the Rule of Law, in Europe, Central Asia and North America in the period 2009–2019.

**Figure:**
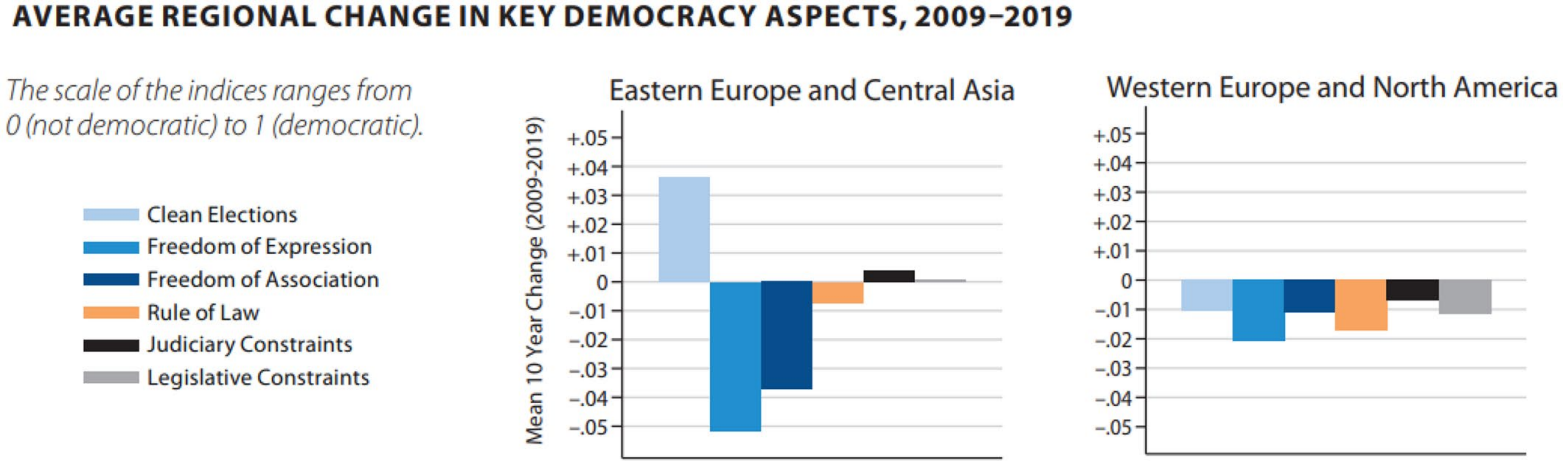
Source: V-DEM Annual Democracy Report 2020 (V-Dem Annual Democracy Report 2020 ‘Autocratization Surges–Resistance Grows’, noted above, at page 19)

Finally, the latest report notes that there is a continuation of trends identified in several previous editions of the Democracy Report, including when it comes to varying democratic component indices. The graph below taken from that report shows this trend, with indices below the diagonal line—including the Rule of Law—indicating that more countries registered negative changes than improved.

**Figure:**
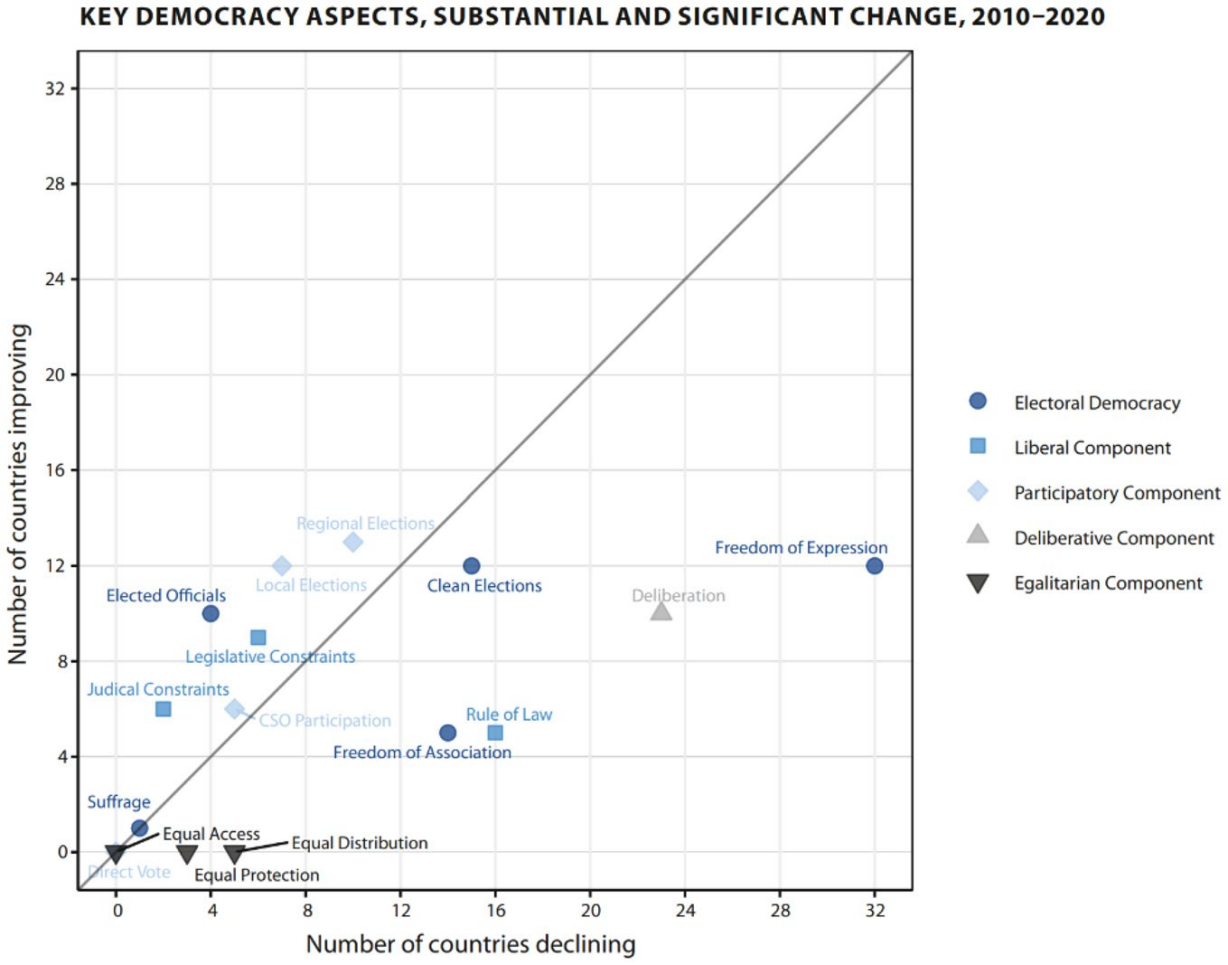
Source: V-DEM Annual Democracy Report 2021 (V-Dem Annual Democracy Report 2021 ‘Autocratization Turns Viral’, noted above, at page 24)

### The Democracy Barometer

The Democracy Barometer, noted above, sponsored by the Swiss National Foundation, consists of a total of 100 indicators, most of which are based on objectively measurable statistics and representative surveys (rather than expert assessments based on subjective evaluations, which are often debatable and not transparent). As discussed above, the Democracy Barometer comprises a Rule of Law component which measures aspects such as equality before the law (i.e. impartial courts, effective independence of the judiciary and effective impartiality of the legal system) and the quality of the legal system (i.e. judicial professionalism and confidence in the justice system and in the police).^129^ Data from 53 countries shows a modest decrease of the global average for equality before the law between 2000 and 2016 (from 50.93 to 45.23) and a more notable decrease in the EU average in the same period (from 62.98 to 54.73).^130^ Interestingly, the Barometer shows that a sharper drop occurred in older EU member states (from an average score of 75.35 in 2000 to 65.42 in 2016) compared to new EU member states, though they also experienced a significant decline. At the national level, the indicator fell sharply in Italy, Bulgaria and Slovakia but notably improved in Serbia, for instance.

### The Bertelsmann Stiftung’s Transformation Index (BTI)

The Bertelsmann Stiftung’s Transformation Index (BTI) analyses and evaluates whether and how developing countries and countries in transition are progressing toward democracy and a market economy. Methodologically, country experts in the 137 countries covered by the index assess the extent to which a total of 17 criteria have been met. The BTI then aggregates the results into two indices: first, the Status Index, with its two analytic dimensions of political and economic transformations in the path toward democracy, the Rule of Law and a social market economy; and second, the Governance Index, which assesses the quality of political leadership that steers transformation processes.

In the BTI, the Rule of Law measure consists of the following elements: separation of powers/checks and balances; judicial independence; prosecution of public officials who abuse their positions; and civil rights protection. RECONNECT research shows that there has been a slight decrease of the average global score [For the Index? For the Rule of Law measure?] between 2006 and 2018, with the most significant drops being recorded in Thailand (− 3.5), Hungary (− 3), and Mozambique (− 2.75). Notable improvements occurred in Liberia (+ 2.5), Bhutan (+ 2.25), and Tunisia (+ 2.25).^131^

The recent BTI 2020 shows a problematic record in the last decade, where the separation of powers has weakened in 60 states, for example in Poland, Romania and Serbia.^132^ As evidenced in the figure below, besides separation of powers, other important declines between 2018 and 2020 were recorded in respect of freedom of expression and association and assembly rights.

**Figure:**
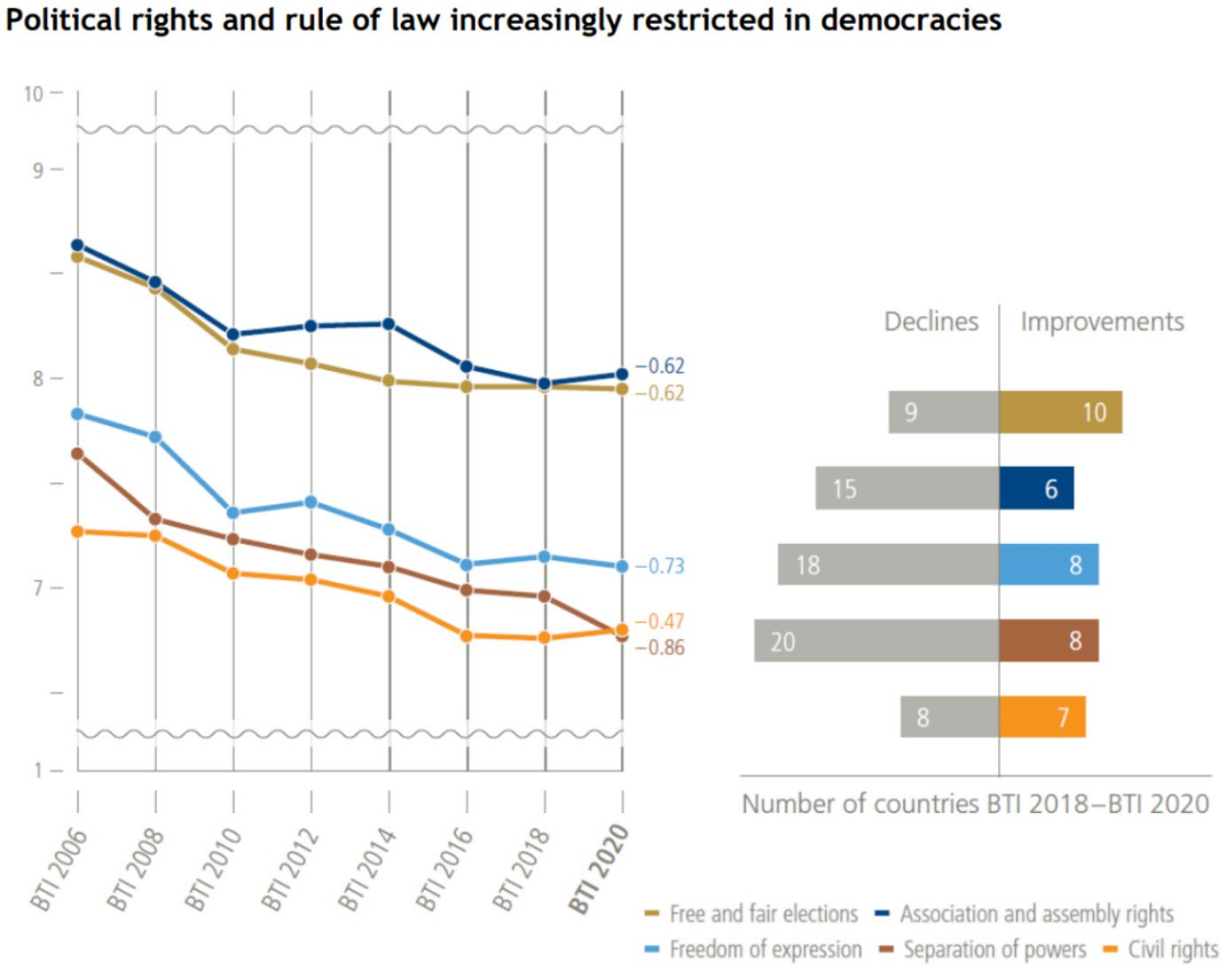
Source: BTI 2020 (BTI 2020, Sabine Donner, Resistance to democratic regression and authoritarian rule is growing: Global Findings Democracy, available at: https://www.bti-project.org/content/en/reports/global-report-d/global_findings_democracy_2020_EN.pdf)

### The World Justice Project’s Rule of Law Index

The World Justice Project’s Rule of Law Index covers 128 countries and jurisdictions, and relies on more than 130,000 household surveys at the national level and on 4,000 specialist assessments by legal practitioners and experts. It measures how the Rule of Law is both experienced and perceived worldwide. The index is comprised of eight factors, namely: constraint on government powers; absence of corruption; open government; fundamental rights; order and security; regulatory enforcement; civil justice; and criminal justice. These are further disaggregated into 44 sub-factors.

The main message from the WJP Rule of Law Index 2021 is that “[d]eterioration in rule of law is spreading worldwide”.^133^ The report shows that “[m]ore countries declined than improved in overall rule of law performance for the fourth consecutive year. In a year dominated by the global COVID-19 pandemic, 74.2% of surveyed countries experienced declines in rule of law performance, while only 25.8% improved. The 74.2% of countries that experienced declines this year account for 84.7% of the world’s population, or approximately 6.5 billion people”.^134^

This trend of a negative direction or of a lack of improvement in Rule of Law performance has affected every region since 2019.

Globally, the biggest improvement in the overall Rule of Law score in the 2021 Index was recorded in Moldova (3.2% score change) gaining 16 places in the global ranking. The biggest declines took place in Belarus (− 7.5% in overall score and − 23% in Factor1, Constraints on Government Powers), in Myanmar (− 6.3%) and in Uzbekistan (− 4.1%). Among EU countries, the biggest decline was recorded in Poland (− 2.4% in overall score and − 6.4% in Factor1, Constraints on Government Powers), followed by Hungary (− 1.4%), Bulgaria (− 1.0%), Croatia (− 1.0%), France (− 0.9%) and Romania (− 0.8%). Other notable mentions are the United States (− 2.9% in overall score and − 4.5% in Factor1, Constraints on Government Powers—the biggest decline in the region) and Turkey (− 2.5%).

Over the last five years (since 2015), the biggest declines regarding constraint on government powers were recorded in Poland (− 6.8%), Hungary (− 5.3%), Serbia (− 6.2%), Bulgaria (− 3.4%), Bosnia and Herzegovina (-5.4%), Albania (− 4.7%) and Turkey (− 4.7%). Some of these countries have also experienced a decline in their overall Rule of Law scores, both in the last year and over the last five years.

**Figure:**
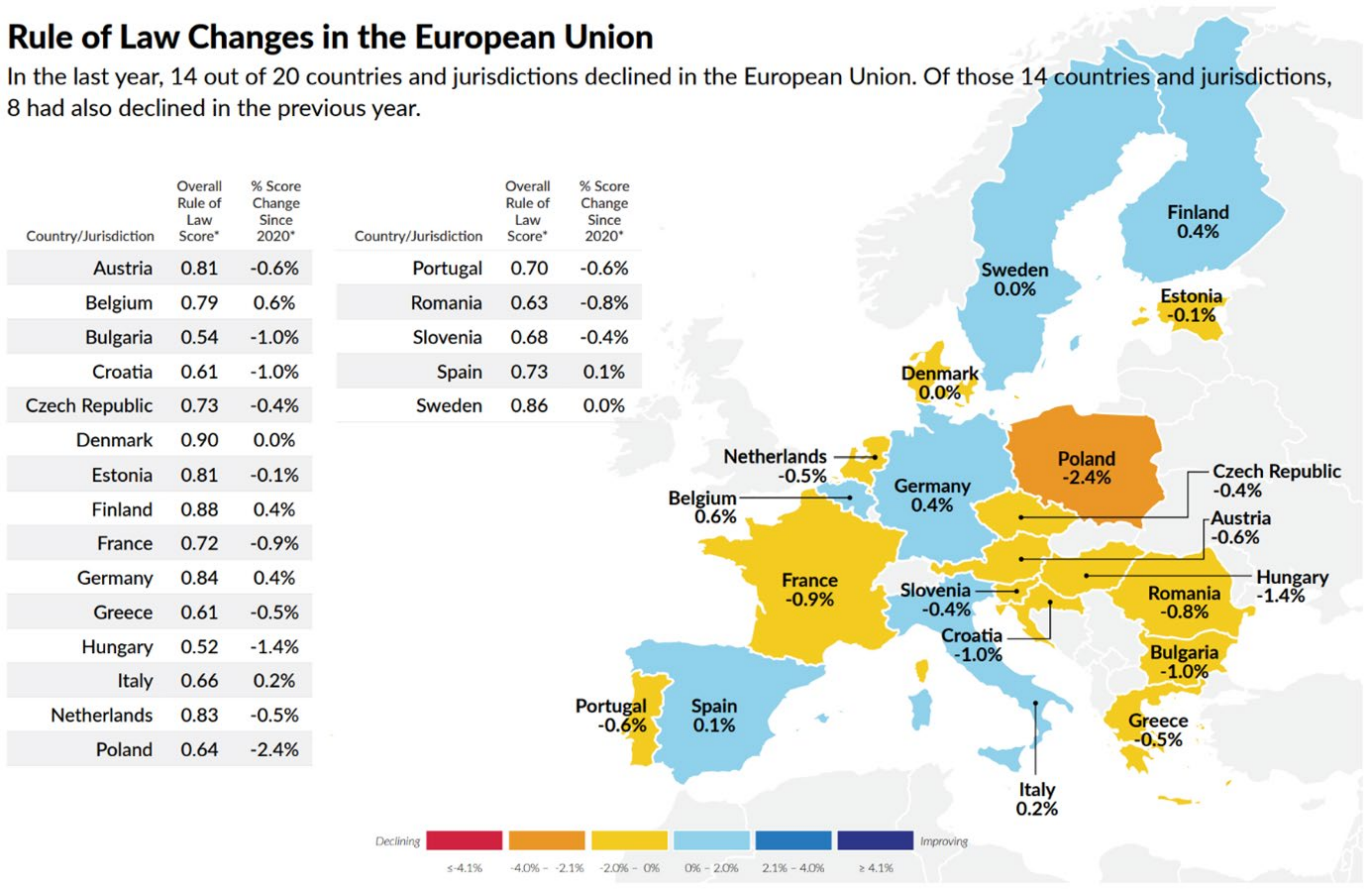
Source: Rule of Law Index 2021 (World Justice Project, Rule of Law Index 2021, Insights Highlights and data, at page 32, available at:  https://worldjusticeproject.org/sites/default/files/documents/WJP-INSIGHTS-21.pdf .)

## Concluding Remarks

Notwithstanding the above-mentioned differences between the various indices in terms of concept specification, type of data used, and aggregation level, it is possible to discern certain recurring trends. Such differences between indices would rather strengthen the validity of common patterns, where detected.

One common finding of the different indices is the gradual decline of the Rule of Law in countries across the world over recent years. This trend has become particularly concerning and apparent in the 2021 measurements. A second recurring message is that Europe is not exempt from Rule of Law weakening and stagnation. A third highlight is that Rule of Law progress is often slow and inconsistent. Nevertheless, certain countries appear consistently across the different indices showing declining Rule of Law patterns.

Indices and the common patterns that may be drawn from them are particularly useful for an objective and comparative assessment of evolutions and trends over time, and even for signalling, in certain cases, an upcoming Rule of Law crisis. While moments that shake the stability of the Rule of Law happen rapidly, the ingredients of the crisis would have been fermenting for some time. It is important to capture those signals, whether through quantitative measurements (where possible) or through qualitative assessments of country case studies.

The COVID-19 pandemic has raised many challenges for the Rule of Law and for efforts to realise the 2030 Agenda for Sustainable Development. It has also impacted on the availability of data for measuring progress towards the SDGs. Investment, innovation and capacity building are needed to respond to such data challenges in order, as put in the 2021 Sustainable Development Goals Report, to “save lives and build back better”.^135^

## Footnotes

1 See discussion in Venice Commission, ‘Report on the Rule of Law’, 2011, CDL-AD(2011)003rev-e, at paras 34, 68–69, available at  www.venice.coe.int/webforms/documents/?pdf=CDL-AD(2011)003rev-e.

2 For further discussion, see e.g., ‘Unity and Diversity in National Understandings of the Rule of Law in the EU’, RECONNECT Work Package 7 – Deliverable 1, 30 April 2020, available at  https://reconnect-europe.eu/wp-content/uploads/2020/05/D7.1-1.pdf > and ‘Meaning and Scope of the EU Rule of Law’, RECONNECT Work Package 7 – Deliverable 2, 30 April 2020, available at < https://reconnect-europe.eu/wp-content/uploads/2020/05/D7.2-1.pdf.

3 T Bingham, ‘The Rule of Law’ (Penguin Books, 2011), at page 8.

4 Bingham identifies the following eight principles: The Accessibility of the Law; Law not Discretion; Equality Before the Law; The Exercise of Power; Human Rights; Dispute Resolution; A Fair Trial; and The Rule of Law in the International Legal Order.

5 J Jowell, ‘The Rule of Law’ in J Jowell and C O’Cinneide (Eds), ‘The Changing Constitution’, 9th Edition (Oxford University Press, 2019), at page 10.

6 Ibid, at page 14.

7 For further discussion, see P Craig, ‘Formal and substantive conceptions of the rule of law: an analytical framework’, Public Law 1997, Aut, 467–487. See also N Barber, ‘Must Legalistic Conceptions of the Rule of Law Have a Social Dimension?’, Ratio Juris 2004, Vol 17 No 4, 474–488; P Rijpkema ‘The rule of law beyond thick and thin’, Law & Philosophy 2013, 32(6), 793–816; and J Waldron, ‘The Rule of Law and the Importance of Procedure’, New York University, Public Law and Legal Theory Research Paper Series, Working Paper No. 10–73, October 2010.

8 Venice Commission, ‘Report on the Rule of Law’, 2011, noted above.

9 Ibid, at para 15.

10 Ibid, at para 41.

11 Ibid, at para 69.

12 Venice Commission, ‘The Rule of Law Checklist’, 2016, available at  https://book.coe.int/en/constitutional-law/7019-pdf-the-rule-of-law-checklist.html.

13 European Commission Communication, ‘A new EU Framework to strengthen the Rule of Law’, 19 March 2014, COM(2014) 158 final/2 (corrected English version), at page 4, available at  https://eur-lex.europa.eu/legal-content/EN/TXT/?uri=CELEX:52014DC0158R(01). See original document 11 March 2014, COM(2014) 158 final, available at  https://eur-lex.europa.eu/legal-content/EN/TXT/?uri=COM:2014:0158:FIN > . See also Annexes 1 to 2, available at  https://data.consilium.europa.eu/doc/document/ST-7632-2014-ADD-1/en/pdf.

14 European Commission Communication, ‘Further strengthening the Rule of Law within the Union: State of play and possible next steps’, 3 April 2019, COM(2019) 163 final, at page 1, available at  https://eur-lex.europa.eu/legal-content/EN/TXT/?uri=CELEX:52019DC0163.

15 Ibid.

16 Report of the Secretary-General, ‘The rule of law and transitional justice in conflict and post-conflict societies’, 23 August 2004, S/2004/616, at para 6, available at   http://undocs.org/en/S/2004/616.

17 Ibid. See also UN website https://www.un.org/ruleoflaw/what-is-the-rule-of-law/ .

18 Report of the Secretary-General, ‘Measuring the effectiveness of the support provided by the United Nations system for the promotion of the rule of law in conflict and post-conflict situations’, 11 June 2013, S/2013/341, at para 6, available at http://undocs.org/en/S/2013/341

19 GA Resolution, ‘Declaration of the high-level meeting of the General Assembly on the rule of law at the national and international levels’, 30 November 2012, A/RES/67/1, at para 2, available at < http://undocs.org/en/A/RES/67/1.

20 M Versteeg and T Ginsburg, ‘Measuring the Rule of Law: A Comparison of Indicators’, Law & Social Inquiry 2017, Vol 42 No 1, 100–137. See also S Skaaning, ‘Measuring the Rule of Law’, Political Research Quarterly 2010, Vol 63 No 2, 449–460.

21 DPKO and OHCHR, ‘The UN Rule of Law Indicators: Implementation Guide and Project Tools’, 2011, available at   https://www.un.org/en/events/peacekeepersday/2011/publications/un_rule_of_law_indicators.pdf . These are complemented by UNDP ‘Why, what and how to measure? A user's guide to measuring rule of law, justice and security programmes’, 2015, available at < https://www.undp.org/content/undp/en/home/librarypage/crisis-prevention-and-recovery/why--what-and-how-to-measure--a-users-guide-to-measuring-rule-of.html.

22 Ibid, at page v.

23 See UN Statistics Division website < https://unstats.un.org/home/faq/ > .

24 See, for example, A Jakab & L Kirchmair, ‘How to Develop the EU Justice Scoreboard into a Rule of Law Index: Using an Existing Tool in the EU Rule of Law Crisis in a More Efficient Way’, German Law Journal 2021, Vol 22 No 6, 936–955; and S Merry, K Davis and B Kingsbury (Eds), ‘The Quiet Power of Indicators: Measuring Governance, Corruption, and Rule of Law’ (Cambridge Studies in Law and Society) (Cambridge University Press 2015). See also, the special issue and the editorial by J Botero, R Nelson and C Pratt, ‘Indices and indicators of justice, governance and the rule of law: an overview’, Hague Journal on the Rule of Law 2011, Vol 3 No 2, 153–169; and T Ginsburg, ‘Pitfalls of measuring the rule of law’, Hague Journal on the Rule of Law 2011, Vol 3 No 2, 269–280.

25 ‘World Justice Project Rule of Law Index 2020’, at page 9, available at < https://worldjusticeproject.org/sites/default/files/documents/WJP-ROLI-2020-Online_0.pdf.

26 See https://democracybarometer.org. The other eight components are individual liberties, public sphere, competition, mutual constraints, governmental capability, transparency, participation and representation.

27 ‘Democracy Barometer Codebook Version 7’, August 2020, at page 16, available at  https://democracybarometerorg.files.wordpress.com/2020/11/codebook_1990-2017_version_homepage.pdf.

28 See N Weinberg, ‘Rule of Law Measurement: Uses and Users’, Background paper for the 5 February 2020 roundtable, on file with the authors.

29 For further discussion, see for example, J Parsons, ‘Developing clusters of indicators: an alternative approach to measuring the provision of justice’, Hague Journal on the Rule of Law 2011, Vol 3 No 2, 170–185. See also S Skaaning, ‘Different Types of Data and the Validity of Democracy Measures’, Politics and Governance 2018, Vol 6 No 1, 105–116; and Vera Institute of Justice, ‘Developing Indicators to Measure the Rule of Law: A Global Approach’, 2008, available at  https://www.vera.org/publications/developing-indicators-to-measure-the-rule-of-law-a-global-approach.

30 N Weinberg, ‘Chasing reality: Rule of Law measurement is lagging years behind current developments’, Verfassungsblog, 30 July 2020, available at  https://verfassungsblog.de/chasing-reality/.

31 Ibid.

32 See N Weinberg, ‘Rule of Law Measurement: Uses and Users’, above.

33 See CEPEJ activities and outputs here: https://www.coe.int/en/web/cepej/home.

34 CEPEJ-GT-EVAL activities and reports are available here:  https://www.coe.int/en/web/cepej/cepej-work/evaluation-of-judicial-systems

35 The CEPEJ dynamic database of European judicial systems can be accessed here: https://www.coe.int/en/web/cepej/cepej-stat.

36 Venice Commission Study No. 494/2008, ‘Report on the Independence of the Judicial System—Part 1: the Independence of Judges’, (CDL-AD(2010)004-e), 16 March 2010, at para. 52.

37 European judicial systems, CEPEJ Evaluation Report, Part 1—Tables, graphs and analyses, September 2020, https://rm.coe.int/evaluation-report-part-1-english/16809fc058, at page 70.

38 The Clearance Rate (CR) is defined as the ratio obtained by dividing the number of resolved cases by the number of incoming cases in a given period, expressed as a percentage. See CEPEJ, Explanatory Note to the Scheme for Evaluating Judicial Systems, 2020—2022 Cycle, CEPEJ(2020)18rev, at page 37.

39 The Disposition Time (DT) is defined as the ratio between pending cases and resolved cases (in days). It shows the theoretical duration for a court to solve all the pending cases. See CEPEJ, Explanatory Note to the Scheme for Evaluating Judicial Systems, 2020—2022 Cycle, CEPEJ(2020)18rev, at page 38.

40 Pending cases are cases which remain to be resolved by the court concerned at a given point in time. See CEPEJ, Explanatory Note to the Scheme for Evaluating Judicial Systems, 2020—2022 Cycle, CEPEJ(2020)18rev, at page 38.

41 The CEPEJ collects data in particular on the number of pending cases that are older than two years, which is the threshold for the ‘reasonable time of proceedings’ in Article 6§1 of the Convention, indicated by the European Court of Human Rights. See CEPEJ, Explanatory Note to the Scheme for Evaluating Judicial Systems, 2020—2022 Cycle, CEPEJ(2020)18rev, at page 41.

42 European judicial systems, CEPEJ Evaluation Report, Part 1—Tables, graphs and analyses, September 2020, https://rm.coe.int/evaluation-report-part-1-english/16809fc058, at page 107.

43 Venice Commission, ‘The Rule of Law Checklist’, 2016, noted above, at para 25.

44 T Bingham, ‘The Rule of Law’ (Penguin Books, 2011), at page 8, noted above.

45 Venice Commission, ‘Report on the Rule of Law’, 2011, noted above.

46 Ibid, at para 41.

47 Venice Commission, ‘The Rule of Law Checklist’, 2016, noted above, at para 26.

48 Ibid, at para 24.

49 This was covered under “Prohibition of arbitrariness” in the 2011 Report. Ibid.

50 This was covered under “Respect for human rights” and “Non-discrimination and equality before the law” in the 2011 Report. Ibid.

51 This was covered under “Access to justice before independent and impartial courts, including judicial review of administrative acts” in the 2011 Report. Ibid.

52 Venice Commission, ‘The Rule of Law Checklist’, 2016, noted above, at para 27.

53 See for instance CJEU, Case T- 240/16, Judgment of 11 July 2018, § 63; Case T-245/15, Judgment of 8 November 2017, at para 74.

54 F Mauri, ‘The Contribution of the Venice Commission to the Strengthening of the Rule of Law in Europe’, PhD dissertation, Université Côte d’Azur; Università degli studi di Milano—Bicocca, 2021, at page 140, available at < https://tel.archives-ouvertes.fr/tel-03369681/document , citing S Bartole, ‘International Constitutionalism and Conditionality – The Experience of the Venice Commission’, Rivista AIC, vol. 4, 2014.

55 Venice Commission, ‘The Rule of Law Checklist’, 2016, noted above, at para 28.

56 L Pech, J Grogan, et al., ‘Meaning and Scope of the EU Rule of Law’, RECONNECT Working Paper Deliverable 7.2, 30.04.2020, at page 8, available at < https://reconnect-europe.eu/wp-content/uploads/2020/05/D7.2-1.pdf.

57 See for instance below in the context of the EU Justice Scoreboard and the Rule of Law Report.

58 The EU Justice Scoreboard reports are available here  https://ec.europa.eu/info/policies/justice-and-fundamental-rights/upholding-rule-law/eu-justice-scoreboard_en.

59 See, for example, the 2021 EU Justice Scoreboard report at page 1.

60 For 2021 see Flash Eurobarometer 489 ‘Perceived independence of the national justice systems in the EU among the general public’ and Flash Eurobarometer 490 ‘Perceived independence of the national justice systems in the EU among companies’.

61 In 2020, however, the World Economic Forum did not publish the Global Competitiveness Index (GCI) rankings due to the COVID-19 pandemic.

62 EU Justice Scoreboard 2021 < https://ec.europa.eu/info/sites/default/files/eu_justice_scoreboard_2021.pdf.

63 Ibid.

64 European Commission, Eurobarometer on the Rule of Law, July 2019, available at < https://europa.eu/eurobarometer/surveys/detail/2235.

65 See European Commission, ‘Special Eurobarometer 489, Report: Rule of Law’ (July 2019) available at < https://europa.eu/eurobarometer/surveys/detail/2235, at page 1.

66 Ibid, at page 1.

67 Ibid, at pages 3–4.

68 See summary of results on European Commission website < https://ec.europa.eu/info/policies/justice-and-fundamental-rights/upholding-rule-law/rule-law/initiative-strengthen-rule-law-eu_en.

69 Ibid.

70 Ibid.

71 Ibid.

72 Communication from the Commission to the European Parliament, The European Council, The Council, The European Economic and Social Committee and the Committee of the Regions, ‘Strengthening the Rule of Law within the Union. A Blueprint for Action’, Brussels, 17.7.2019 (COM(2019) 343 final).

73 Communication from the Commission to the European Parliament, the European Council and the Council Further strengthening the rule of law within the Union – State of play and possible next steps, Brussels, 3.4.2019 (COM(2019)163 final), noted above.

74 European Commission Communication, ‘Strengthening the Rule of Law within the Union. A Blueprint for Action’, Brussels, 17.7.2019, noted above, at page 5.

75 Ibid, at pages 9–13.

76 Ibid, at pages 9–13.

77 Ibid, at page 9.

78 Ibid, at page 10.

79 Ibid, at page 10.

80 Ibid, at page 11.

81 Communication from the Commission to the European Parliament, the Council, the European Economic and Social Committee and the Committee of the Regions, 2020 Rule of Law Report, The Rule of Law Situation in The European Union, COM/2020/580 final.

82 See P Bárd, B Grabowska-Moroz et al., ‘The strategies and mechanisms used by national authorities to systematically undermine the Rule of Law and possible EU responses’, RECONNECT Work Package 8 – Deliverable 2, 31 December 2020, at page 49, available at  https://reconnect-europe.eu/wp-content/uploads/2021/01/D8.2.pdf

83 Ibid.

84 Communication from the Commission to the European Parliament, the Council, the European Economic and Social Committee and the Committee of the Regions, 2021 Rule of Law Report, The Rule of Law Situation in The European Union, COM/2021/700 final.

85 Ibid, at 3.1.

86 Ibid, at 3.1.

87 Ibid, at 5.

88 See SDGs website  https://www.un.org/sustainabledevelopment/development-agenda/.

89 GA Resolution ‘Transforming Our World: the 2030 Agenda for Sustainable Development’, 21 October 2015, A/RES/70/1, available at  http://undocs.org/en/A/RES/70/1.

90 The 2030 Agenda for Sustainable Development, above, at para 35.

91 See IAEG-SDGs website  https://unstats.un.org/sdgs/iaeg-sdgs/  and UN Statistics Division website < https://unstats.un.org/home/faq/

92 GA Resolution ‘Work of the Statistical Commission pertaining to the 2030 Agenda for Sustainable Development’, 10 July 2017, A/RES/71/313, at para 1, available at < http://undocs.org/en/A/RES/71/313.

93 GA Resolution A/RES/71/313, above.

94 See J Beqiraj, ‘Future perfect: measuring access to justice in the post-2015 development framework’, Public Law 2016, Oct, 546–555, at 551. Comments were made in relation to a previous, almost identical iteration of the global indicators.

95 See the 2020 Comprehensive Review Proposals website  https://unstats.un.org/sdgs/iaeg-sdgs/2020-comprev/UNSC-proposal/. 

96 See official list on the SDG indicators website  https://unstats.un.org/sdgs/indicators/indicators-list/.

97 See for example ‘16.3.3 Indicator Proposal: Access to Civil Justice’, 2019, submitted by two co-custodian agencies, UNDP and OECD, and developed with technical support from OSJI and WJP, available at  https://worldjusticeproject.org/our-work/publications/working-papers/access-civil-justice-indicator-proposal-sdg-target-1633

98 J Botero, A Pinzon-Rondon and C Pratt, ‘How, When and Why Do Governance, Justice and Rule of Law Indicators Fail Public Policy Decision Making in Practice?’, Hague Journal on the Rule of Law 2016, Vol 8 No 1, 51–74, at Sect. 4.4.

99 Ibid.

100 GA Resolution A/RES/71/313, above, at para 1.

101 See Eurostat website  https://ec.europa.eu/eurostat/web/sdi

102 P Gennari and D Kalamvrezos Navarro, ‘Guest Article: Are We Serious About Achieving the SDGs? A Statistician’s Perspective’, IISD SDG Knowledge Hub, 14 January 2020, available at  http://sdg.iisd.org/commentary/guest-articles/are-we-serious-about-achieving-the-sdgs-a-statisticians-perspective/

103 See HLPF website  https://sustainabledevelopment.un.org/hlpf and the 2030 Agenda for Sustainable Development, above, at para 83.

104 Report of the Secretary-General ‘Progress towards the Sustainable Development Goals’, 30 April 2021, E/2021/58, available at  http://undocs.org/en/E/2021/58. 

105 Report of the Secretary-General ‘Progress towards the Sustainable Development Goals: Supplementary Information—Statistical Annex: Global and regional data for Sustainable Development Goal indicators’, [E/2021/58], available at  https://unstats.un.org/sdgs/files/report/2021/secretary-general-sdg-report-2021--Statistical-Annex.pdf .

106 ‘The Sustainable Development Goals Report 2021’, available at  https://unstats.un.org/sdgs/report/2021/

107 In September 2021, the new ‘Global SDG Indicators Data Platform’ was launched which includes a new, user-friendly interface to the Database – see  https://unstats.un.org/sdgs/ . The Platform is available to view here  https://unstats.un.org/sdgs/unsdg

108 ‘The Sustainable Development Goals Report 2021’, above, at page 62.

109 ‘The Sustainable Development Goals Report 2021’, above, at page 62 and the SG Progress Report 2021, above, summary.

110 See UN Statistics Division website, Frequently Asked Questions on ‘SDG monitoring—Stakeholders’, available at < https://unstats.un.org/home/faq/.

111 See IAEG-SDGs website < https://unstats.un.org/sdgs/iaeg-sdgs/tier-classification/ ..

112 International Institute for Sustainable Development (IISD), ‘Tracking the Trackers: SDG Data Sources at Year Five’, IISD SDG Knowledge Hub, 23 July 2020, available at < http://sdg.iisd.org/commentary/policy-briefs/tracking-the-trackers-sdg-data-sources-at-year-five/ > .

113 See IISD, ‘SDG Implementation and Accountability: Bring on the Data’, IISD SDG Knowledge Hub, 15 October 2020, available at < http://sdg.iisd.org/commentary/policy-briefs/sdg-implementation-and-accountability-bring-on-the-data/ > . For example, the Statistical Annex 2021, above, presents indicators for which data is available and for Target 16.3 this is limited to indicator 16.3.2, where it is reported that unsentenced detainees as a proportion of the overall prison population is 31% in 2019, slightly higher than in 2015 (30%) (see at page 189).

114 The Sustainable Development Goals Report 2021, above, at page 5.

115 Ibid, at page 5.

116 'The Sustainable Development Goals Report 2020’ available at < https://unstats.un.org/sdgs/report/2020/ > , at page 4.

117 The Sustainable Development Goals Report 2021, above, at page 5.

118 S MacFeely, ‘To keep track of the SDGs, we need a data revolution’, 17 January 2019, available at < https://unctad.org/news/keep-track-sdgs-we-need-data-revolution > .

119 See main website here < https://www.v-dem.net/ > .

120 V-Dem Annual Democracy Report 2021 ‘Autocratization Turns Viral’, available at: https://www.v-dem.net/static/website/files/dr/dr_2021.pdf.

121 See structure of the index here:  https://www.v-dem.net/static/website/img/refs/structurev111.pdf.

122 Asif Efrat, Sivan Hirsch-Hoefler, Amichai Magen (2019), “Working Paper on Global Democracy & Rule of Law Conditions and Trajectories”, Work Package 3 – Deliverable 3.1, at pages 29–30.

123 V-Dem Annual Democracy Report 2021 ‘Autocratization Turns Viral’, noted above, at page 13.

124 V-Dem Annual Democracy Report 2018 ‘Democracy for All?’, at page 80, available at: https://www.v-dem.net/media/filer_public/68/51/685150f0-47e1-4d03-97bc-45609c3f158d/v-dem_annual_dem_report_2018.pdf.

125 V-Dem Annual Democracy Report 2021 ‘Autocratization Turns Viral’, noted above; V-Dem Annual Democracy Report 2020 ‘Autocratization Surges–Resistance Grows’, available at: https://www.v-dem.net/media/filer_public/de/39/de39af54-0bc5-4421-89ae-fb20dcc53dba/democracy_report.pdf; V-DEM Annual Democracy Report 2019 ‘Democracy Facing Global Challenges’, available at: https://www.v-dem.net/media/filer_public/99/de/99dedd73-f8bc-484c-8b91-44ba601b6e6b/v-dem_democracy_report_2019.pdf.

126 V-Dem Annual Democracy Report 2021 ‘Autocratization Turns Viral’, noted above, at page 7.

127 Ibid, at page 19.

128 Ibid.

129 Democracy Barometer Codebook, August 2020, available at: https://democracybarometerorg.files.wordpress.com/2020/11/codebook_1990-2017_version_homepage.pdf.

130 Asif Efrat, Sivan Hirsch-Hoefler, Amichai Magen (2019), “Working Paper on Global Democracy & Rule of Law Conditions and Trajectories”, Work Package 3 – Deliverable 3.1, at pages 30 ff.

131 Ibid.

132 BTI 2020 Infographics, available at:  https://www.bti-project.org/en/press.html ; BTI 2020, Allan Sikk, Trouble at the Top, Regional Report East-Central and Southeast Europe, available at:  https://www.bti-project.org/content/en/reports/regional-reports/regional_report_ESE_2020_EN.pdf.

133 World Justice Project Rule of Law Index, 2021, available at: https://worldjusticeproject.org/our-work/research-and-data/wjp-rule-law-index-2021.

134 Ibid.

135 The Sustainable Development Goals Report 2021, above, at pages 4–7.

